# Anisotropic Etching of InGaN Thin Films with Photoelectrochemical Etching to Form Quantum Dots

**DOI:** 10.3390/ma16051890

**Published:** 2023-02-24

**Authors:** Xiongliang Wei, Syed Ahmed Al Muyeed, Haotian Xue, Jonathan J. Wierer

**Affiliations:** 1 Center for Photonics and Nanoelectronics, Department of Electrical and Computer Engineering, Lehigh University, Bethlehem, PA 18015, USA; 2 Department of Electrical and Computer Engineering, North Carolina State University, Raleigh, NC 27695, USA

**Keywords:** InGaN, quantum dots, photoelectrochemical etching, anisotropic etching

## Abstract

Traditional methods for synthesizing InGaN quantum dots (QDs), such as the Stranski-Krastanov growth, often result in QD ensembles with low density and non-uniform size distribution. To overcome these challenges, forming QDs using photoelectrochemical (PEC) etching with coherent light has been developed. Anisotropic etching of InGaN thin films is demonstrated here with PEC etching. InGaN films are etched in dilute H_2_SO_4_ and exposed to a pulsed 445 nm laser with a 100 mW/cm^2^ average power density. Two potentials (0.4 V or 0.9 V) measured with respect to an AgCl|Ag reference electrode are applied during PEC etching, resulting in different QDs. Atomic force microscope images show that while the QD density and sizes are similar for both applied potentials, the heights are more uniform and match the initial InGaN thickness at the lower applied potential. Schrodinger-Poisson simulations show that polarization-induced fields in the thin InGaN layer prevent positively charged carriers (holes) from arriving at the c-plane surface. These fields are mitigated in the less polar planes resulting in high etch selectivity for the different planes. The higher applied potential overcomes the polarization fields and breaks the anisotropic etching.

## 1. Introduction

Epitaxial InGaN quantum dots (QDs) could help produce higher-efficiency visible laser diodes (LDs) and light-emitting diodes (LEDs) [1]. InGaN QDs are also interesting for high-speed LEDs for communications [2,3] and single photon sources [4,5]. QDs should have higher spontaneous recombination and optical gain from the well-known benefits of its delta-like density of states [6]. Traditionally, InGaN QDs are formed by self-assembled growth methods, such as Stranski-Krastanov (SK) growth via metalorganic chemical vapor deposition [7,8,9], molecular beam epitaxy [10,11], or DC magnetron sputtering [12,13]. SK growth relies on the spontaneous formation of QDs due to the strain of the underlying growth layers. It is the typical growth method used to create InGaN-based LEDs and LDs [11,14,15,16]. It is challenging with SK to synthesize InGaN QDs with uniform size distribution because of the spontaneous formation of the QDs. Therefore, new approaches are needed to produce QDs with high density and controlled sizes to enable the potential benefits of QDs.

An alternative method to create InGaN QDs is to use quantum-sized-controlled photoelectrochemical (PEC) etching [17,18,19]. This method can overcome the size and density limitations of self-assembled growth. In this method, PEC etching of InGaN thin films on GaN is performed using a coherent (laser) light source. The etching proceeds by absorption of the laser light only in the InGaN layer, and the additional electrolyte and applied potential cause etching via oxidation of InGaN and dissolution of this oxide layer. As the QDs become small enough, the etch self-terminates because the QD absorption energies shift, so they can no longer absorb the laser light. InGaN QDs that emit in the violet-blue have been formed using this method; they have densities and sizes that rival self-assembled QDs [20]. However, they only emit at cryogenic temperatures (5 K), most likely due to the high defect and surface recombination on the exposed QD surfaces [21]. Growing passivation layers on top enable room temperature luminescence, but the efficiency is still too low to compete with the efficiencies of InGaN quantum wells [21].

Although the PEC etched QDs are not ideal light emitters on their own, they can be used as template layers to achieve control of SK QD growth. It has been demonstrated that PEC-etched quantum dots as growth templates can positively influence Stranski-Krastanov growth [20,22]. The PEC QD templates are created by, first, PEC etching to form InGaN QDs, and then, growing thin AlGaN/GaN capping layers. The AlGaN/GaN thicknesses are chosen to cover the PEC QDs, but also kept thin enough so that the strain of the PEC QDs is still present in the capping layer to influence the growth of subsequently grown SK QDs. Additionally, the AlGaN layer is at a high Al-content that prevents the decomposition of the InGaN QDs [22]. The SK QDs vertically align to and take on the dimensional properties of the PEC QDs due to localized strain. QDs formed this way are smaller sized than the traditional SK QDs and have high dot densities. This structure enables multiple quantum dot layers that are not exposed and are subject to surface recombination. They have also been successfully used to demonstrate InGaN QD LEDs that emit at near-IR wavelengths [22].

One limitation of the PEC-etched QDs as growth templates is that they tend to be of different heights. The growth of the SK QDs on top of the PEC QDs (stacking) relies on the strain of the PEC QDs to be present at the AlGaN/GaN capping layer surfaces. Therefore, some of the PEC QDs are of the right height and ensure the SK QDs stack. However, the shorter PEC QDs are not tall enough to induce strain on the surface, and so do not seed SK QD growth. This will lead to a loss in the SK QD density compared to the PEC QD template. Ensuring high QD densities is necessary for the performance of QD emitters.

This paper adds to previous reports and demonstrates that anisotropic PEC etching with a coherent light source to form InGaN QDs can be achieved under the proper etch conditions. The mechanisms of the PEC etching under these anisotropic etch conditions are explored. The InGaN films are etched in dilute H_2_SO_4_ and exposed to a pulsed 445 nm laser. Two potentials with respect to the AgCl|Ag reference electrode (0.4 V or 0.9 V) are applied during PEC etching, resulting in a different QD formation. At a higher potential, the QDs have different heights; at a lower potential, the QDs are the same height and match the initial film thickness. The QDs consistent height results from polarization-induced fields in the thin InGaN layer that prevent positively charged carriers (holes) from arriving at the *c*-plane surface. These fields are not present at less polar planes. This results in greater positive charge and faster in-plane etching of the less polar planes. The higher potential overcomes this limitation and breaks the anisotropic etching.

## 2. Materials and Methods

InGaN/GaN thin films used for PEC etching are grown using metalorganic chemical vapor deposition (MOCVD) in a Veeco P-75 reactor. The layers are grown on *c*-plane templates consisting of Si-doped, n-type GaN on patterned sapphire substrates. The GaN template layer has an electron concentration of ~3 × 10^18^ cm^−3^ and a thickness of ~4 μm. On this template, an n-type GaN layer is grown with a thickness of ~100 nm and an electron concentration of ~10^18^ cm^−3^. Unintentionally doped (no dopant added during growth) and ~6–8 nm thick In_y_Ga_1−y_N thin films are then grown at 730 °C, resulting in y~0.15. A III-V ratio of 43,000 is used, producing relatively smooth films with high radiative efficiency. The InGaN layers emit at ~460 nm under photoluminescence with a 405 nm laser.

**Figure 1:**
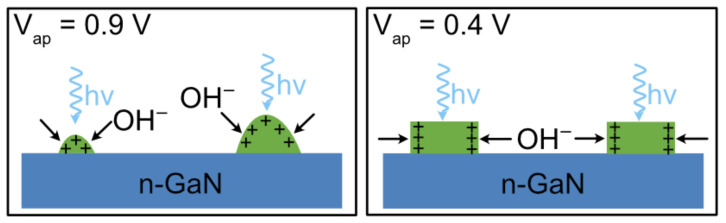
Cross-sectional schematics of the PEC etch process with a coherent light source with two different applied potential (V_ap_). The InGaN layer (green) absorbs the light, creating electrons and hole pairs, where the holes collect at the surface. Anisotropy in the distribution of holes creates anisotropic etching that depends on the applied potential.

These InGaN thin films are then subject to photoelectrochemical (PEC) etching with a coherent light source to create QDs. The InGaN films are etched in an electrochemical cell with an applied potential with respect to the reference electrode of 0.4 V or 0.9 V (Figure 1) controlled by a potentiostat with a Pt electrode and an Ag|AgCl reference electrode. An evaporated Al contact is formed on the InGaN/GaN sample and rests outside the electrolyte. The electrolyte consists of 0.2 M dilute H_2_SO_4_ with a pH of ~0.4. The sample is illuminated with a 445 nm pulsed laser (5% duty cycle, 20 kHz) with an average power density of 100 mW/cm^2^_._ Pulsing of the laser provides the required oxidation and reduction etching of the InGaN [21]. The laser light is diffused using an optical diffuser to create a lit area of 1.5 cm × 1.5 cm. The samples are stopped over time to investigate the evolution of the QD formation. The etch is considered completed when the current is less than 2 μA, but QDs are formed at higher currents. During etching, the current flowing through the electrolyte/semiconductor is recorded.

The InGaN thin films are also subject to impedance spectroscopy and Mott-Schottky analysis to determine the flat-band voltage and the electron concentrations in the semiconductor layers [23]. The impedance spectroscopy measurement is also performed in the electrochemical cell with the same electrolyte—but without the laser light. The DC potential is applied to the sample with a small AC signal that is varied in frequency (10–10^4^ Hz). At each frequency, the impedance is measured. The impedance versus angular frequency (Bode plot) is fitted using a simple model consisting of a capacitor and resistor series to determine the capacitance, C, expressed as

(1) $$\frac{1}{C^{2}}=\frac{2}{q\varepsilon_{s}N_{D}A^{2}}\left(V_{ap}-V_{FB}-\frac{kT}{q}\right),$$

where *A* is the etched area, *V_ap_* is the applied potential between the sample and reference electrode (Ag|AgCl), *V_FB_* is the flat band potential, and *N_D_* is the donor concentration of the semiconductor. This is repeated at various applied DC potentials. The Mott-Schottky analysis is performed by plotting 1/*C*^2^ versus the applied DC potential. The slope of the lines is used to determine the electron concentration in the different layers as the depletion width changes with the applied potential. The location where the data crosses the x-axis provides the built-in potential caused by band bending in the semiconductor at the electrolyte/semiconductor interface.

The PEC-etched QDs are subject to atomic force microscopy (AFM) using a Veeco DI 5000 in tapping mode with a high-resolution Si tip with a tip radius of 1 nm. The AFM images are used to determine the evolution of the PEC etch process and the dimensions of the final QD ensembles. The AFM data are visualized and analyzed using the Gwyddion software package (version 2.6) [24].

Schrodinger-Poisson simulations using nextnano^++^ solver (version 1.10.19–2022.080101) [25] are performed to determine the band diagrams of the InGaN/GaN heterostructures. These band diagrams illustrate how band bending due to polarization fields results in anisotropic etching. The PEC etching at a low potential is modeled using modified photoconductivity equations that consider the changing surface morphology and anisotropic etching.

## 3. Results and Discussion

The results section is divided into PEC etch results and modeling. First, impedance spectroscopy and Mott-Schottky analysis are used to determine the built-in potential caused by the electrolyte/semiconductor interface. Then PEC etching is performed at two different applied potentials to cause different band bending and carrier types at the semiconductor surface. The two potentials result in different forms of quantum dots. The etching results are understood by modeling band diagrams using Schrodinger-Poisson simulations and a simple photoconductive etch model.

### 3.1. Photoelectrochemical Etching Results

**Figure 2:**
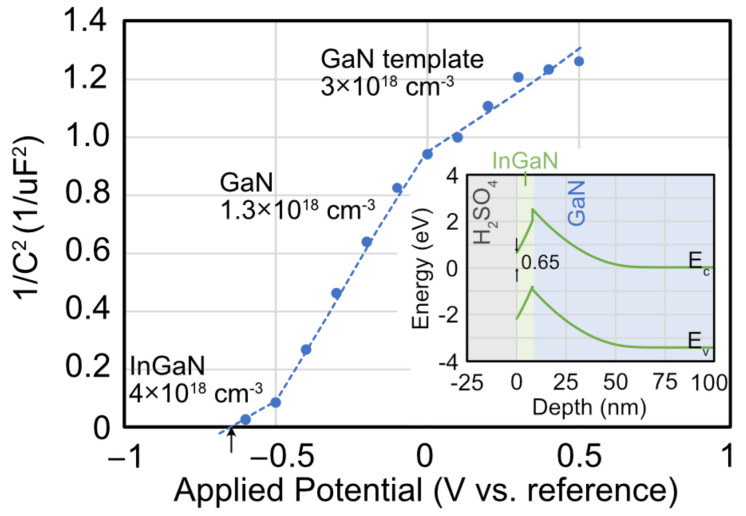
The inverse of the capacitance squared (1/C^2^) versus applied potential with respect to the AgCl|Ag reference electrode is determined by impedance spectroscopy. The three fitted lines determine the electron concentration in the InGaN, GaN, and GaN template layers. The data crosses the x-axis at −0.65 V (arrow), which is the flat-band potential at the electrolyte/semiconductor interface. The inset shows the band diagram for the H_2_SO_4_/InGaN/GaN structure along the *c*-direction at equilibrium with zero applied potential. The y-axis is positioned, so the Fermi-energy level in the GaN template is at 0 eV.

The InGaN/GaN thin films are subject to impedance spectroscopy and Mott-Schottky analysis to determine the built-in potential and confirm the doping in the different layers. Figure 2 shows a plot of the inverse of the capacitance squared versus the applied potential between the sample and the reference electrode. Three different lines are fit to the data and represent the three different layers in the epitaxial structure. The ranges of potentials match the targeted thicknesses as the depletion width sweeps through the sample. At the top is the unintentionally doped InGaN layer, which has an electron concentration of 4 × 10^18^ cm^−3^. The 100 nm thick GaN layer is next, with an electron concentration of 1.4 × 10^18^ cm^−3^, slightly higher than the targeted 10^18^ cm^−3^. Finally, at the bottom is the template GaN layer with an electron concentration of 3 × 10^18^ cm^−3^. The flat-band potential (V_FB_) is found where the line crosses the x-axis at −0.65 V. To create flat-band conditions, an applied potential of V_FB_ = −0.65 V is required.

The built-in potential gives a basic understanding of the band diagram of the InGaN/GaN structure shown for the c-direction in the inset of Figure 2. (Note: the Helmholtz layer at the GaN surface is considered small and ignored.) The InGaN layer has an energy bandgap of ~2.7 eV and an intrinsic (mid-gap) energy of 1.35 eV. The y-axis is positioned, so the Fermi-energy level in the GaN template is at 0 eV. It can be recalibrated to the reversible hydrogen electrode using the Nernst equation, which includes the standard redox potential and the pH. Polarization charges cause band bending at the GaN/InGaN interface that does not change with applied potential. The high electron concentration causes the Fermi energy level to be close to the conduction band so that the barrier height can be approximated as 0.65 V. The etch potentials of 0.4 V and 0.9 V will bend the bands further up so that the differences in the conduction band and intrinsic energy (E_c_–E_i_) are 1.05 eV and 1.55 eV, respectively. Therefore, at V_ap_= 0.4 V in the electrochemical cell, the surface will be slightly n-type. At V_ap_= 0.9 V, the semiconductor surface will be slightly p-type. It will be shown below that the applied potential affects the measured current during etching and the resulting height of the quantum dots.

**Figure 3:**
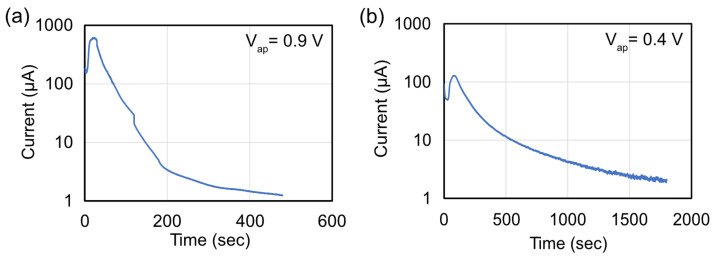
Etch current versus time at V_ap_ of (**a**) 0.9 V and (**b**) 0.4 V. (The discontinuities in (**a**) are caused by stopping the etch to measure AFMs.) At 0.9 V, the etch current has a higher peak and is completed in a shorter amount of time.

Figure 3 shows the etch currents at the two different potentials. The currents at V_ap_ = 0.9 V have a higher peak current, and the InGaN etches faster than at V_ap_ = 0.4 V. (Note: the discontinuities in the V_ap_ = 0.9 V curve are a result of removing the sample to perform AFM. This removal from the electrochemical cell shifts the currents slightly.) The V_ap_ = 0.9 V has a 4.5-times-higher peak current than at V_ap_ = 0.4 V. Some of that higher current can be explained by the higher voltage (2.25 times higher). It will be shown that at 0.4 V, the etch is anisotropic, and this anisotropy is reduced at 0.9 V. So, another difference in the current is caused by the availability of the different etch planes and a larger area to etch.

**Figure 4:**
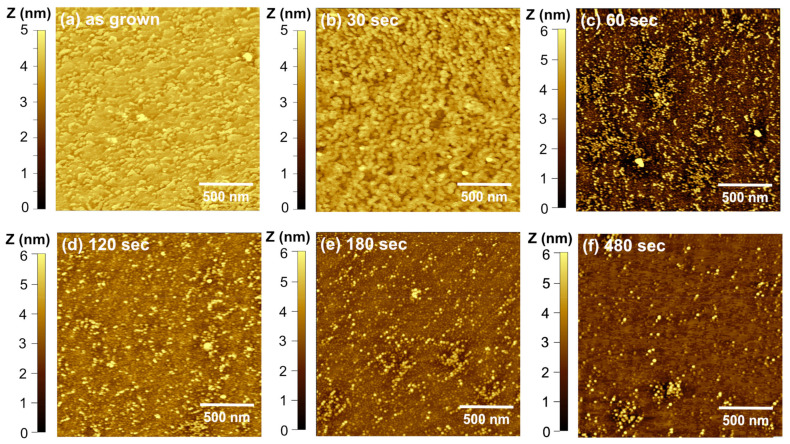
Atomic force microscope images showing the progress of InGaN QD formation using PEC etching with a coherent light source at 0.9 V. The images are for the (**a**) as-grown surface and etched for (**b**) 30 s, (**c**) 60 s, (**d**) 120 s, (**e**) 180 s, and (**f**) 480 s. The QDs form at 60 s and have heights that vary from 2 to 5 nm. As the etching time increases, the QD density decreases from ~7 × 10^10^/cm^2^ at 60 s to ~1.5 × 10^10^/cm^2^ at 480 s.

Figure 4 shows AFM images at different times for an InGaN/GaN sample etched at a V_ap_ = 0.9 V. In the initial stages (30 s), the surface becomes rougher and distinct structures begin to form. They appear to be collections of circular shapes in string-like formations that tend to follow the surface atomic steps of the underlying GaN. This morphology is also viewed at 0.4 V, which eventually determines the quantum dots’ location. This formation may be caused by the strong polarization-induced fields induced by the strain that repels carriers away from the GaN atomic steps. At 60 s, the current decreases exponentially and quantum dots begin to form. For this time, the dot density is high at ~7 × 10^10^ cm^−2^, and the QDs are different heights. Given the inhomogeneities in the InGaN compositions [25], the etch may first attack the higher In-content areas where the absorption is higher. Additionally, the high applied potential significantly increases the etching preference difference and causes a more significant variation in QDs size. As the etching progresses, the dot density is reduced as some smaller dots are etched away. At 480 s, the density of dots is ~1.5 × 10^10^ cm^−2^, and the heights range from 2–5 nm. The QD heights are smaller than the initial thickness of the InGaN layer at ~7 nm. Although some etch selectivity causes QD formation, etching along all crystal directions is still present under these etch conditions.

**Figure 5:**
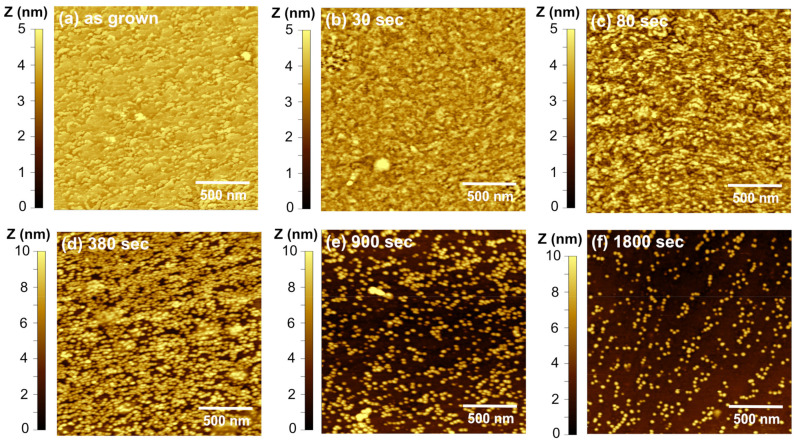
Atomic force microscope images exhibiting the progress of InGaN QD formation using PEC etching with a coherent light source at 0.4 V. The images are for the (**a**) as-grown surface and etched for (**b**) 30 s, (**c**) 80 s, (**d**) 380 s, (**e**) 900 s, and (**f**) 1800 s. The QDs formation is slower than at 0.9 V, and the QDs are fully formed at 380 s. QD density reduces from ~1 × 10^11^/ cm^2^ at 380 s to ~1.2 × 10^10^/cm^2^ at 1800 s.

Lowing the etch potential results in more uniform QD heights caused by highly anisotropic etching. Figure 5 shows AFM images at different times for an InGaN/GaN sample etched at V_ap_ = 0.4 V. Similar to the higher potential sample, there is a roughening of the surface as the etch current increases up to 80 s, resulting in similar string-like formations. At 380 s, quantum dots are formed with an extremely high dot density of ~10^11^ cm^−2^. As etching proceeds, the density decreases and is 1.2 × 10^10^/cm^2^ at 1800 s. There is a marked difference in the QD heights at this lower applied potential. The QDs retain the same height as the original film thickness at ~7 nm. This suggests a high anisotropy in etch process, where in-plane etching is preferred compared to the *c*-direction (vertical). After the initial stages, where the etch roughens the surface, the heights of the QDs do not change with further etching. Only the dot density decreases, as seen when comparing Figure 5d,e. The anisotropic etch is attenuated by raising the potential to 0.9 V. This suggests that positive charge accumulation, which is required for etching, is different on the different crystal planes at 0.4 V. This is supported by band structure modeling below.

**Figure 6:**
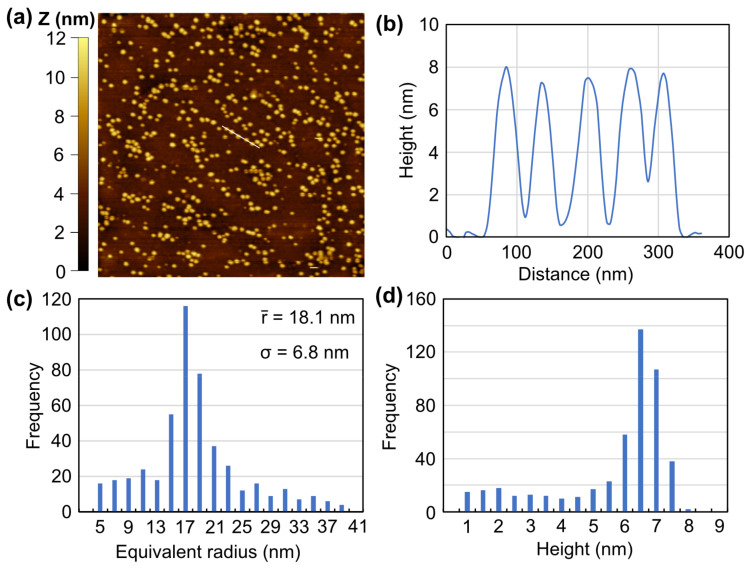
Atomic force microscope image (**a**) of QDs etched at 0.4 V for 30 min, similar to Figure 4f. A plot (**b**) of height versus distance for the cross-sectional line (white) in (**a**). Frequency versus bins of QD equivalent radius (**c**) and height (**d**) for the image shown in (**a**). The QDs have a tight distribution for radius with a mean value of 18.1 nm. The QD heights are concentrated at ~7.5 nm, the initial InGaN thin film thickness.

Figure 6 shows an InGaN/GaN sample etched similarly to Figure 5 at a potential of 0.4 V. The AFM (Figure 6a) shows that the QDs are a similar height. This is confirmed by the line scan in Figure 6b, indicating that the QDs have heights of ~7.5 nm. The image in Figure 6a is analyzed; histograms of the equivalent radius and QD heights are shown in Figure 6c,d, respectively. The QD radiuses are in a tight distribution with a mean radius of 18.1 nm and a standard deviation of 6.8 nm. This is similar to previous reports of PEC etched QDs [20]. These dimensions are much smaller in size and tighter distribution that can be achieved with SK growth in the same reactor (mean of 37.8 nm and standard deviation of 17.8 nm) [20]. They are also tighter in distribution than SK growth of InGaN QDs that were analyzed in other reports [26,27]. The heights are concentrated at ~7–7.5 nm, with a slight tail to smaller heights showing that the etch anisotropy is not perfect. Some of the distribution in height can be attributed to the thickness uniformity of the initial film (see Figure 4a, for example) and the non-planarity of the GaN template. Shorter QDs could be achieved using this anisotropic etch if the initial InGaN layer was thinner.

The anisotropic etching demonstrated in Figure 5 and Figure 6 results in QDs that are nearly the same height. Therefore, the height of the QDs is controlled by the thin-film epitaxial growth, which can be very precise. The lateral dimensions are determined by the in-plane etching. Compared to QDs formed by SK growth, which are typically much wider than taller (~20–40 nm wide and ~3–5 nm tall), the anisotropic PEC etching provides ultimate height control and some control of the overall QD shape. The densities achieved here are on the order of 10^10^–10^11^ cm^−2^, which are similar to SK growth [14,28]. Therefore, the major benefit of PEC etched QDs is dimensional control, especially when used as templates for SK growth.

The reactivity of the different surfaces could also play a role in the etch selectivity. Highly crystallographic or anisotropic etching has been shown using Tetramethylammonium hydroxide (TMAH), H_3_PO4, and KOH of GaN [29,30,31]. H_2_SO_4_ is used here, which did not exhibit etch selectivity in previous reports [31]. Therefore, the primary anisotropic etch mechanism is most likely caused by the polarization fields within the crystal.

### 3.2. Photoelectrochemical Etching Model

**Figure 7:**
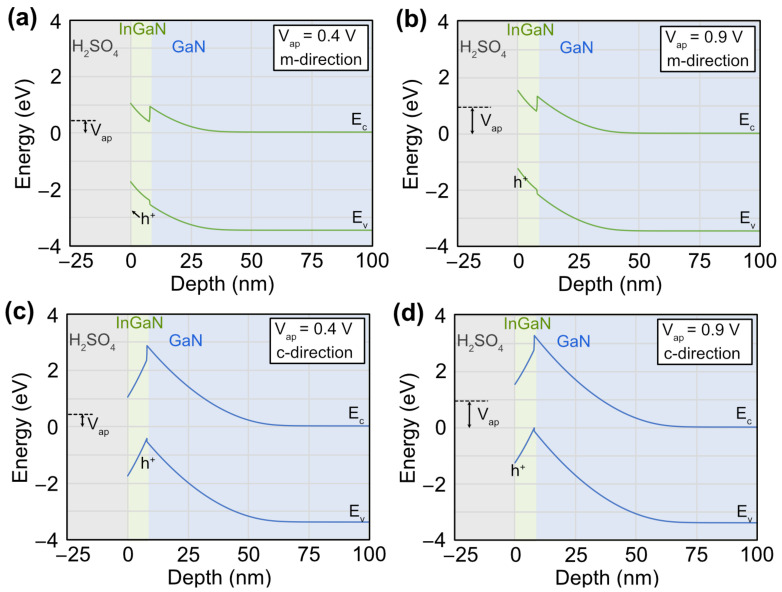
Simulated band diagram for the InGaN/GaN structure at applied potentials versus the reference electrode and directions of (**a**) 0.4 V and (**b**) 0.9 V for the *m*-direction, and (**c**) 0.4 V and (**d**) 0.9 V for the *c*-direction. The dotted line is the energy of the reference electrode. The polarization fields create a well at the InGaN/GaN interface for the *c*-direction, where holes can collect (**d**). At a potential of 0.9 V, the InGaN surface is p-type, and the applied potential alone promotes etching and allows for both orientations to etch. At a potential of 0.4 V, the InGaN surface is n-type, and holes generated by the absorbed light are easier to collect on the *m*-plane surface than the *c*-plane. The y-axis is positioned, so the Fermi-energy level in the GaN template is at 0 eV, but it can be recalibrated to the reversible hydrogen electrode using the Nernst equation.

The anisotropic etching can be explained by investigating the band diagrams determined from Schrodinger-Poisson simulations. Figure 7 shows the band diagrams for applied potentials and directions of (a) 0.4 V and (b) 0.9 V for the *m*-direction, and (c) 0.4 V and (d) 0.9 V for the *c*-direction. The Fermi energy is nearly equal to the conduction band energy in the neutral region, so the barrier height is set at −V_FB_ =0.65 V. The *c*-direction has large piezoelectric and spontaneous polarization that causes electric fields and severe band bending of the InGaN layer (upward into the structure). This causes a potential well for holes at the InGaN/GaN heterointerface. This potential well can inhibit holes created by the absorption of the laser light to travel to the surface and promote etching. At 0.4 V, the surface potential is 1.05 V. The InGaN layer has a bandgap of ~2.8 eV, so the surface is n-type. In the *m*-direction, where the polarization fields are not present, the holes can travel to the surface, but this is not possible in the *c*-direction. The n-type c-plane surface will not have the positive charge required for etching. Hence, high anisotropy in etching is observed.

At a potential of 0.9 V, the surface potential is 1.55 V. This means that the surface of the InGaN is p-type. Therefore, the applied potential alone can provide a positive charge to promote etching on the *c*-plane. The potential well is still present, so one should expect a difference in change and etch rate depending on the direction. There should also be some anisotropy at these higher potentials but with a lower selectivity than observed at lower potentials.

**Figure 8:**
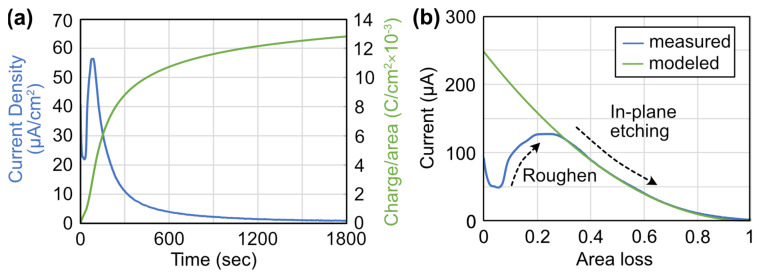
Plot of (**a**) current density and charge density versus time for an 8 nm thick InGaN layer at a potential of 0.4 V. The charge density is used to calculate the amount of InGaN volume etched over time. Plot of (**b**) measured and modeled current versus InGaN area loss at 0.4 V. At the initial stages of etching, the current increases due to surface roughening. The current can be modeled at higher area loss with a modified photoconductivity model.

The etching mechanism can be explained using a simple photoconductive model. Figure 8a shows the etch current density versus time for an 8 nm thick InGaN layer at a potential of 0.4 V. This current can be converted to charge density assuming the number of electrons transferred per unit InGaN is 3 [17]. This charge density versus time can be further converted into the volume of InGaN consumed versus time using the expression

(2) $$volume=A\left(t-\sigma /qQ\right),$$

where *A* is the lit area, *t* is the initial layer thickness, *σ* is the etched charge per area (C/cm^2^), and *Q* is the charge density of InGaN. Anisotropic etching means that the volume loss can be translated into a fractional area loss (AL). The current is replotted versus area loss in Figure 8b and shown with the blue curve.

At the beginning of the etch process, the InGaN film is roughened, and the exposed surface area increases, leading to an increase in the etch current. Once the less polar planes are exposed, the current is dominated by in-planed etching that can be modeled with photoconductive current equations [32] that are modified to include the *AL*. The current is modeled using equations for generation rate, *G*, efficiency, *η*, and current, ∆*I* expressed as

(3) $$G=\frac{\eta P}{h\nu A}\left(AL\right)\;,$$

(4) $$\eta =\eta_{i}\left(1-R\right)\left(1-e^{-\alpha t}\right)\left(AL\right),\;\mathrm{and}$$

(5) $$\Delta I=q\mu_{n}G\tau_{n}V_{ap}\left(A/t\right),$$

where *P* is the average laser power, *η_i_* (0.8) is the internal quantum efficiency, *R* surface power reflection, *α* (10^−2^ /cm) absorption coefficient of the InGaN film at the laser wavelength, *μ_n_* (200 cm^2^/V-s) mobility of electrons, and *τ_n_* (10^−7^ s) lifetime of electrons. The current is a drift current dominated by the more mobile electrons. The fit is excellent for the exponential decay of the curve.

Modeling the current at a potential of 0.9 V from Figure 2 with Equations (3)–(5) does not result in a good fit. At 0.4 V, the etch current is only along the m-planes, and the InGaN etched can be expressed as an area loss parameter. When the potential becomes 0.9 V the etch is isotropic, and the c-plane is etched, too. Therefore, the etch model cannot be simplified by using an area loss parameter.

This fit provides some insights into the etching, foremost of which is that using InGaN films with low defect recombination rates is critical to forming QDs. To quantify the defect recombination rate, the following calculation is undertaken. The InGaN film in Figure 8 is very thin (~8 nm) and the laser light is near the band edge, resulting in a low generation rate of ~5.5 × 10^13^ /cm^3^-s at the peak current in Figure 8b. At this generation rate, the recombination will be dominated by defect and possibly spontaneous (band-to-band) recombination. During the laser pulse, one can assume steady state, and the generation rate will be equal to the recombination rates expressed as

(6) $$G=AN+BN^{2}$$

where *N* is the electron concentration, *A* the coefficient for defect recombination, and *B* is the coefficient for spontaneous emission. The two terms on the right-hand side represent the defect and spontaneous emission rate. For an InGaN layer emitting at 460 nm, the typical A and B coefficients are 10^7^ s^−1^ and 5 × 10^−12^ cm^3^/s, respectively [33]. Using these values, the carrier density and recombination rates can be determined. The carrier density is ~10^8^ cm^−3^; the defect recombination rate is nearly the same as the generation rate. This means that the recombination of the electron-hole pairs is dominated by defect recombination in these films. Films with a greater number of defects (higher A coefficients) will result in shorter recombination lifetimes and lower etch currents (see Equation (4)). This will result in difficulties in QD formation. Indeed, InGaN films emitting at green wavelengths with higher A coefficients (<10^7^ s^−1^) and lower internal quantum efficiency have difficulty forming quantum dots.

Finally, the data above shows a switch from isotropic to anisotropic etching when changing the etch potential. One hypothesis is that there is a threshold voltage where, at low potential, the etching is anisotropic. When increasing the voltage, a threshold is then reached analogous to the turn-on in a semiconductor p-n junction (diode). The voltage changes the hole concentration at the surface exponentially, leading to higher etch currents and isotropic etching. Exploring this hypothesis and the etch results at different voltages are left for future work.

## 4. Conclusions

Anisotropic etching of InGaN layers to form quantum dots using PEC etching with a laser light source is demonstrated. At a low enough applied potential, holes created by the absorption of the laser light are prevented from accumulating on the *c*-plane surface because of large polarization fields creating a well at the InGaN/GaN heterointerface. Therefore, in-plane etching—where the polarization fields are weaker—dominates. This results in QD heights that match the thickness of the original InGaN film. The etching results in dot densities that are high, >10^10^/cm^−2^, and radiuses that are smaller, <20 nm, which can be achieved with traditional self-assembled growth methods.

## Data Availability

The data presented in this study are available on request from the corresponding author.
